# Isolated limb perfusion with melphalan activates interferon-stimulated genes to induce tumor regression in patients with melanoma in-transit metastasis

**DOI:** 10.1080/2162402X.2019.1684126

**Published:** 2019-11-03

**Authors:** Junko Johansson, Roberta Kiffin, Ebru Aydin, Malin S. Nilsson, Kristoffer Hellstrand, Per Lindnér, Peter Naredi, Roger Olofsson Bagge, Anna Martner

**Affiliations:** a TIMM Laboratory, Sahlgrenska Cancer Center, Sahlgrenska Academy, University of Gothenburg, Gothenburg, Sweden; b Department of Surgery, Institute of Clinical Sciences, Sahlgrenska Academy, University of Gothenburg, Gothenburg, Sweden; c Department of Infectious Diseases, Institute of Biomedicine, Sahlgrenska Academy, University of Gothenburg, Gothenburg, Sweden; d Transplantation Centre, Sahlgrenska University Hospital, Gothenburg, Sweden; e Department of Surgery, Sahlgrenska University Hospital, Gothenburg, Sweden; f Wallenberg Centre for Molecular and Translational Medicine, University of Gothenburg, Gothenburg, Sweden

**Keywords:** Melanoma, isolated limb perfusion, melphalan, ISG, chemokines, T cell chemotaxis

## Abstract

Hyperthermic isolated limb perfusion (ILP) with high-dose melphalan is a treatment option for melanoma patients with metastasis confined to limbs (in-transit metastasis). The therapy entails a complete response (CR) rate of 50–70%. Cellular immunity is proposed to impact on the clinical efficacy of ILP, but the detailed aspects of ILP-induced immune activation remain to be explored. For this study, we explored the potential role of interferon-stimulated gene (ISG) products, including CXCL10, CCL2, PD-L2 and IFN-γ along with expression of their cognate receptors CXCR3, CCR4, CCR5 and PD-1 on lymphocytes, for the clinical efficacy of ILP. Patients with high serum levels of CXCL10, CCL2, PD-L2 and IFN-γ were more likely to achieve CR after ILP. Additionally, the expression of CXCR3, CCR4 and CCR5 on T cells and/or natural killer (NK) cells was enhanced by ILP. Peripheral blood mononuclear cells (PBMCs) secreted high levels of CXCL10, CCL2 and IFN-γ in response to co-culture with melphalan-exposed melanoma cells *in vitro*. Activated T cells migrated toward supernatants from these co-cultures. Furthermore, melphalan-exposed melanoma cells triggered upregulation of CXCR3, CCR4, CCR5 and PD-1 on co-cultured T cells and/or NK cells. Our results suggest that constituents released from melphalan-exposed melanoma cells stimulate the ISG axis with ensuing formation of chemokines and upregulation of chemokine receptor expression on anti-neoplastic immune cells, which may contribute in ILP-induced tumor regression.

## Introduction

Interferons (IFNs) are potent antimicrobial proteins that control the spread of viral infections but are also endowed with immunomodulatory properties.^1^ Upon ligating their cognate receptors, type I (IFN-α, IFN-β) and type II (IFN-γ) IFNs signal via the JAK-STAT pathway, which may result in the expression of hundreds of interferon-stimulated genes (ISGs).^2^ Several ISGs encode proteins that participate in protection against pathogens, but ISG products are also involved in immune modulation and chemotaxis.^3^

Earlier studies have demonstrated a link between the degree of T cell infiltration in malignant tumors and an intratumoral type I and type II interferon signature.^4^ ISG products thus comprise T cell-recruiting chemokines, including CCL2, CCL4, CCL5 and CXCL10. CXCL10 is primarily induced by IFN-γ in monocytes, endothelial cells and fibroblasts.^5^ Its cognate receptor, CXCR3, is expressed by activated T cells and NK cells^6,7^ but may also be expressed by melanoma cells.^8,9^ CCR4 interacts with CCL2, CCL4 and CCL5, while CCR5 interacts with CCL4 and CCL5. CCR4 and CCR5 may be expressed by T cells and NK cells.

Isolated limb perfusion (ILP) is a procedure that allows for the local administration of systemically intolerable doses of melphalan to an isolated limb. ILP is used primarily in patients with melanoma metastases confined to a limb (in-transit metastasis). The impact of ILP on overall survival has not yet been documented in larger studies; however, ILP carries a complete response (CR) rate, referring to the complete disappearance of tumors after 3 months, of 50–70%.^10,11^ Patients often experience tumor regression during several months after perfusion,^12^ which implies that the treatment may comprise activation of anti-tumoral immunity. In line with this assumption, we have recently reported that ILP triggers an increase of activated T cells in peripheral blood, and that CR following ILP is significantly associated with presence of activated and antigen-specific CD8^+^ T cells prior to perfusion.^13,14^ These findings thus support that induction of cellular immunity may, in part, explain the clinical benefit of ILP.

The purported role of an immune-related mechanism of action of ILP, along with the potential impact of ISGs for the recruitment of T cells to the tumor microenvironment, incited us to investigate a putative link between ILP and ISG products. We assessed systemic levels of ISG-related chemokines and the expression of their cognate receptors on lymphocytes among peripheral blood mononuclear cells (PBMCs) before and after ILP and correlated these aspects of ISG biology with clinical outcome. Our results imply that induction of ISG products and their receptors may contribute to the anti-tumor efficacy of ILP.

## Results

### Association between high expression of ISG products and clinical efficacy of ILP

**Figure 1.**
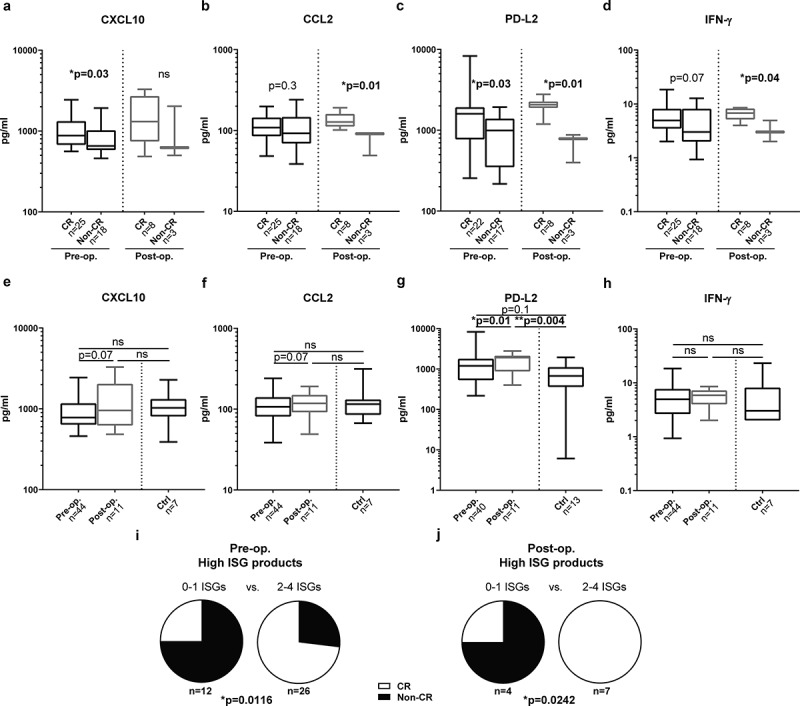
ISG product levels predict CR after ILP. The serum concentration of (a) CXCL10, (b) CCL2, (c) PD-L2 and (d) IFN-γ in melanoma patients achieving a complete response (CR) and not achieving a complete response (Non-CR) before (pre-op.) and 1 month after (post-op.) ILP (non-paired Mann-Whitney test). The serum concentration of (e) CXCL10, (f) CCL2, (g) PD-L2 and (h) IFN-γ in melanoma patients and in healthy controls (Ctrl) (paired Wilcoxon test between pre-op. and post-op., non-paired Kruskal-Wallis test followed by Dunn’s multiple comparison test for ctrl vs. pre-op. and vs. post-op). Data are presented in box-and-whiskers plots with min. and max. (i,j) Patients were dichotomized by above or below median levels of CXCL10, CCL2, PD-L2 and IFN-γ and were grouped based on having above median levels of 0–1 ISG products, or 2–4 ISG products. The fraction of patients within each group achieving CR or not (non-CR) are shown for ISG-grouping based on (i) pre-op. samples (n = 38) and (j) post-op. samples (n = 11; Fisher’s exact test).

Serum samples were collected before ILP (43 patients) and follow-up samples were collected 1 month later (11 patients). Serum was analyzed for content of ISG products such as chemokines and other soluble factors using multiplex immunoassays. Fifty-eight percent of the patients achieved CR following ILP, and the levels of ISG products were compared between CR and non-CR patients. Patients achieving CR showed significantly higher serum levels of CXCL10 and PD-L2 prior to ILP (Figure 1(a,c)) and higher levels of CCL2, PD-L2 and IFN-γ 1 month after ILP (Figure 1(b–d)). Serum levels of PD-L2 increased significantly following ILP with similar albeit non-significant trends for CXCL10, CCL2 and IFN-γ (Figure 1(e–h)). These trends toward ISG induction were not observed for CCL4 or CCL5 (Fig. S1). The serum levels of ISG products were similar in melanoma patients and healthy subjects with the exception of PD-L2 that was higher in melanoma patients after ILP than in control subjects (Figure 1(g)).

Patients were also dichotomized by having above or below median serum levels of none to 1 or 2 to 4 of the ISG products CXCL10, CCL2, PD-L2 and IFN-γ. Patients with above median expression levels of multiple ISG products, before or after ILP, were significantly more likely to achieve CR (Figure 1(i,j)). Additionally, we compared the ISG product levels among patients with different Wieberdink limb toxicity scores^15^ but did not observe correlations between the level of ISG transcription and the toxicity of the ILP regimen (data not shown).

### Intratumoral expression of ISGs and ISG products

Single-cell suspensions from tumor biopsies obtained prior to ILP were available from 10 patients. Supernatants obtained after culture of the single-cell suspensions for 48 h were analyzed for CCL2 and CXCL10 by ELISA, while mRNA purified from the cells in the culture was analyzed for expression of *CCL2, CXCL10* and *PDCD1LG2* by RT-qPCR. These analyzes did not reveal significant differences in ISG expression levels between CR and non-CR patients. Apart from a partial responder (PR) patient (marked by a half-filled circle in the figure), all non-CR patients expressed low levels of ISGs on gene and protein levels compared with CR patients (Fig. S2).

### High ISG expression levels in advanced melanoma tumors are linked to a favorable prognosis

**Figure 2.**
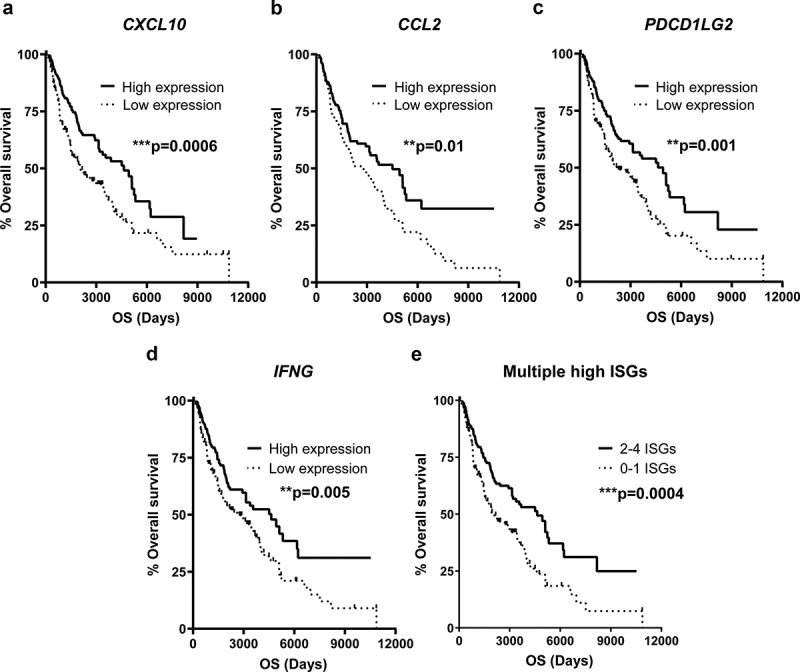
High levels of intratumoral ISG transcripts predict prolonged survival in advanced melanoma. Melanoma patients in the TCGA database were dichotomized by above or below median mRNA expression of the ISGs (a) *CXCL10*, (b) *CCL2*, (c*) PDCD1LG2*, (d*) IFNG* or (e) 0–1 or 2–4 of these ISGs, followed by analysis of overall survival by the log-rank test (n = 470).

The favorable impact of ISG induction, as reflected by the presence of systemic ISG-encoded proteins, on the clinical outcome after ILP incited us to investigate the potential correlation between transcription of ISGs and survival in a larger melanoma dataset. We thus mined The Cancer Genome Atlas (TCGA, http://cancergenome.nih.gov/) for intratumoral ISG mRNA in patients with advanced/metastatic melanoma (n = 470). It was observed that patients with above median intratumoral mRNA expression of *CXCL10, CCL2, PDCD1LG2* or *IFNG* showed significantly improved overall survival (Figure 2(a–d)). The effect on survival was pronounced for patients with above median expression of multiple ISGs (Figure 2(e)).

### ILP induces expression of receptors for ISG products on PBMCs

**Figure 3.**
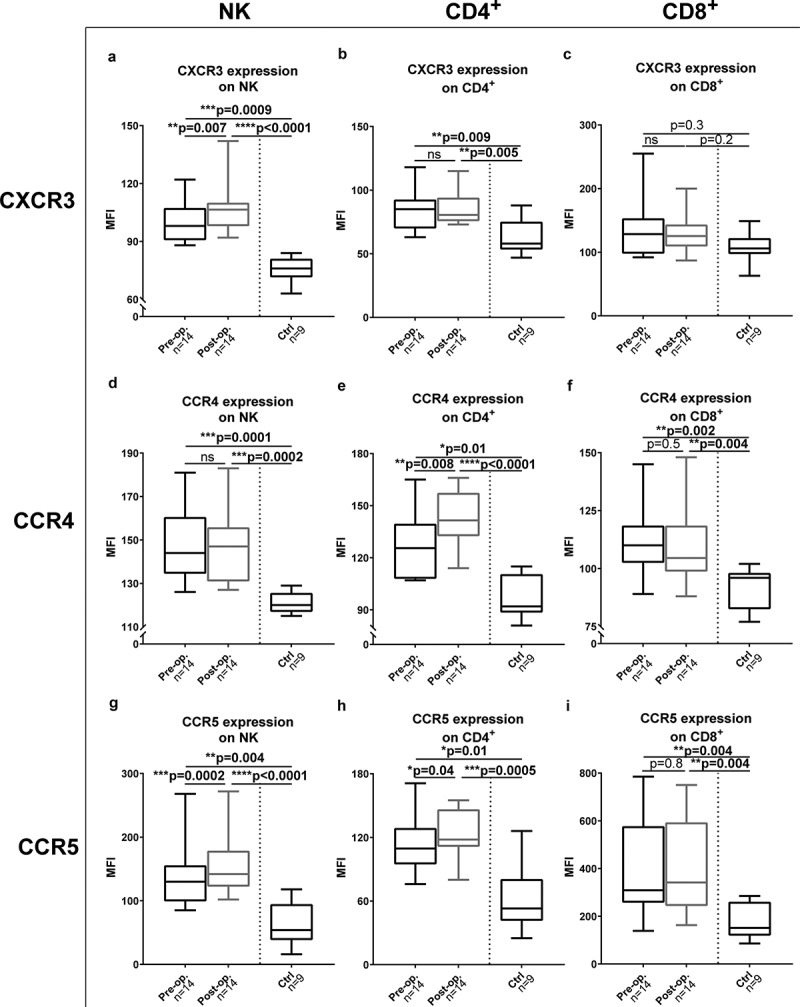
ILP causes induction of receptors for ISG products on PBMCs. The expression of (a–c) CXCR3 (d–f) CCR4 and (g–i) CCR5 were measured on (a, d and g) NK cells (b, e and h) CD4^+^ T cells and (c, f and i) CD8^+^ T cells from melanoma patients before (pre-op.) and 1 month after (post-op.) ILP and from healthy controls (Ctrl) (Paired Wilcoxon test between pre-op. and post-op., non-paired Kruskal-Wallis test followed by Dunn’s multiple comparison test for ctrl vs. pre-op. and vs. post-op). MFI, Median Fluorescence Intensity. Data are presented in box-and-whiskers plots with min. and max.

PBMCs were isolated from peripheral blood from 14 melanoma patients before and after ILP. T cells and NK cells within PBMCs were analyzed by flow cytometry for expression of receptors to ISG products. It was observed that the expression of CXCR3, the cognate receptor to CXCL10, and CCR5, the receptor to CCL4 and CCL5, was significantly increased on NK cells following ILP (Figure 3(a,g)). Furthermore, the expression of CCR4, the receptor to CCL2, CCL4 and CCL5, as well as the expression of CCR5 increased significantly on CD4^+^ T cells after ILP (Figure 3(e,h)). Compared with PBMCs from healthy controls, melanoma patients harbored CD4^+^ T cells and NK cells with higher expression of CXCR3 (Figure 3(a,b)) along with NK cells, CD4^+^ T cells and CD8^+^ T cells with higher expression of the receptors CCR4 and CCR5 (Figure 3(d–i)).

We previously showed that the levels of regulatory T cells (T_regs_) in blood and their expression of PD-1 increases following ILP.^13^ In analyzes aiming to define if the high chemokine receptor expression reflected expression in T_regs_ or in conventional CD4^+^ T cells, it was observed that the expression of CXCR3 was significantly higher on conventional CD4^+^ T cells while T_regs_ (CD3^+^CD4^+^CD25^+^CD127^−^)^16^ expressed higher levels of CCR4 and CCR5 (Fig. S3).

### Receptor expression on intratumoral and peripheral blood lymphocytes

**Figure 4.**
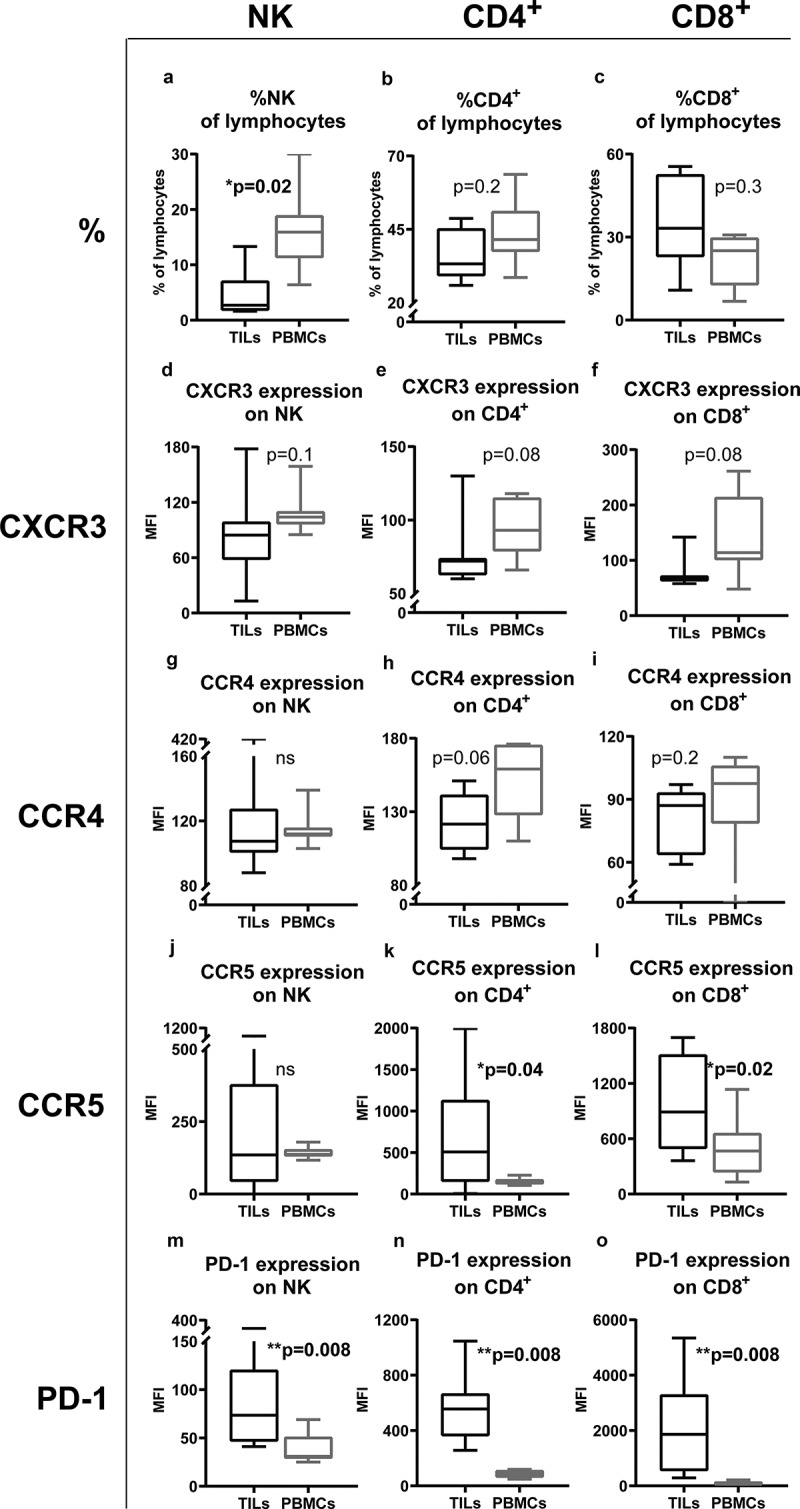
Different expression patterns of receptors on TILs and on PBMCs. The percentage of (a) NK cells, (b) CD4+ T cells and (c) CD8+ T cells among all lymphocytes in tumorbiopsies obtained during perfusion and in peripheral blood taken before ILP. Expression of (d-f)CXCR3, (g-i) CCR4, (j-l) CCR5 and (m-o) PD-1 on (d,g,j,m) NK cells, (e,h,k,n) CD4+ T cellsand (f,i,l,o) CD8+ T cells in tumors (TILs) and in peripheral blood (PBMCs) (n=8; paired Wilcoxon test). MFI = Median Fluorescence Intensity. Data are presented in box-and-whiskers plots with min. and max.

Tumor-infiltrating lymphocytes (TILs) were analyzed from eight tumor biopsies, and the T and NK cell content along with receptor expression of intratumoral lymphocytes were compared with lymphocytes in peripheral blood from the same patients. There was a trend toward a higher fraction of CD8^+^ T cells in tumors while the frequency of NK cells and CD4^+^ T cells among lymphocytes was lower in tumors than in peripheral blood (Figure 4(a–c)). The expression of CCR5 and PD-1 was significantly higher on TILs compared with lymphocytes in blood while the opposite trend was observed for expression of CXCR3 and CCR4 (Figure 4(d–o)). Due to a low number of non-CR patients, analysis of the impact of TILs and their receptor expression on outcome could not be performed. However, chemokine receptor expression by transcriptome analysis in the TCGA melanoma data set revealed that high intratumoral content of CXCR3, CCR5 and PD-1 were significantly beneficial in terms of overall survival (Fig. S4).

### Melphalan-exposed melanoma cells trigger secretion of CXCL10, CCL2 and IFN-γ from PBMCs

**Figure 5.**
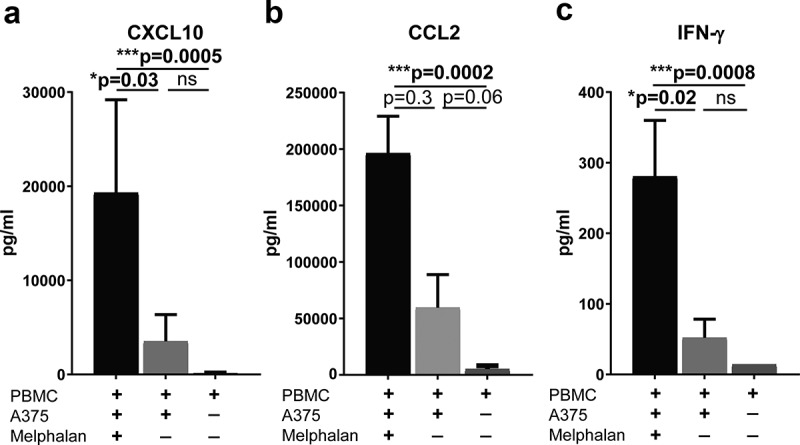
Melphalan-exposed melanoma cells induce expression of CXCL10, CCL2 and IFN-γ in PBMCs during co-culture. PBMCs from healthy donors were cultured with melphalan-exposed melanoma cells, non-exposed melanoma cells or were cultured alone for 48 h. After 48 h the levels of (a) CXCL10, (b) CCL2 and (c) IFN-γ were measured in the cell culture supernatants (n = 9; paired Friedman test followed by Dunn’s multiple comparison test). Data are presented as mean with SEM.

To define the mechanistic impact of melphalan on ISG induction during ILP, an *in vitro* model of ILP was established wherein A375 cells were exposed to a sub-lethal concentration of melphalan and subsequently co-cultured with PBMCs from healthy donors. Cell culture supernatants collected after 48 h of co-culture were analyzed for soluble CXCL10, CCL2, IFN-γ, IFN-α2 and IFN-β. While no detectable levels of IFN-α2 and IFN-β were found, it was observed that melphalan-exposed melanoma cells triggered extensive production of CXCL10, CCL2 and IFN-γ from co-cultured PBMCs (Figure 5(a–c)). Co-cultures of non-exposed melanoma cells with PBMCs showed higher levels of CXCL10, CCL2 and IFN-γ than PBMCs cultured alone, but the levels were considerably lower in the absence of melphalan (Figure 5(a–c)). Melanoma cells cultured alone, with or without melphalan-exposure, produced non-detectable or very low levels of CXCL10 (<20 pg/ml, n = 6), approximately 100 times less CCL2 than the levels in melphalan-exposed co-cultures (1740 ± 370 pg/ml, mean ± SEM, n = 6) and non-detectable levels of IFN-γ (<28 pg/ml). Supernatants obtained from melphalan-exposed melanoma cells did not mimic the ISG-stimulating effect of the melphalan-exposed melanoma cells (data not shown).

### Melphalan-exposed melanoma cells stimulate expression of receptors for ISG products on lymphocytes

**Figure 6.**
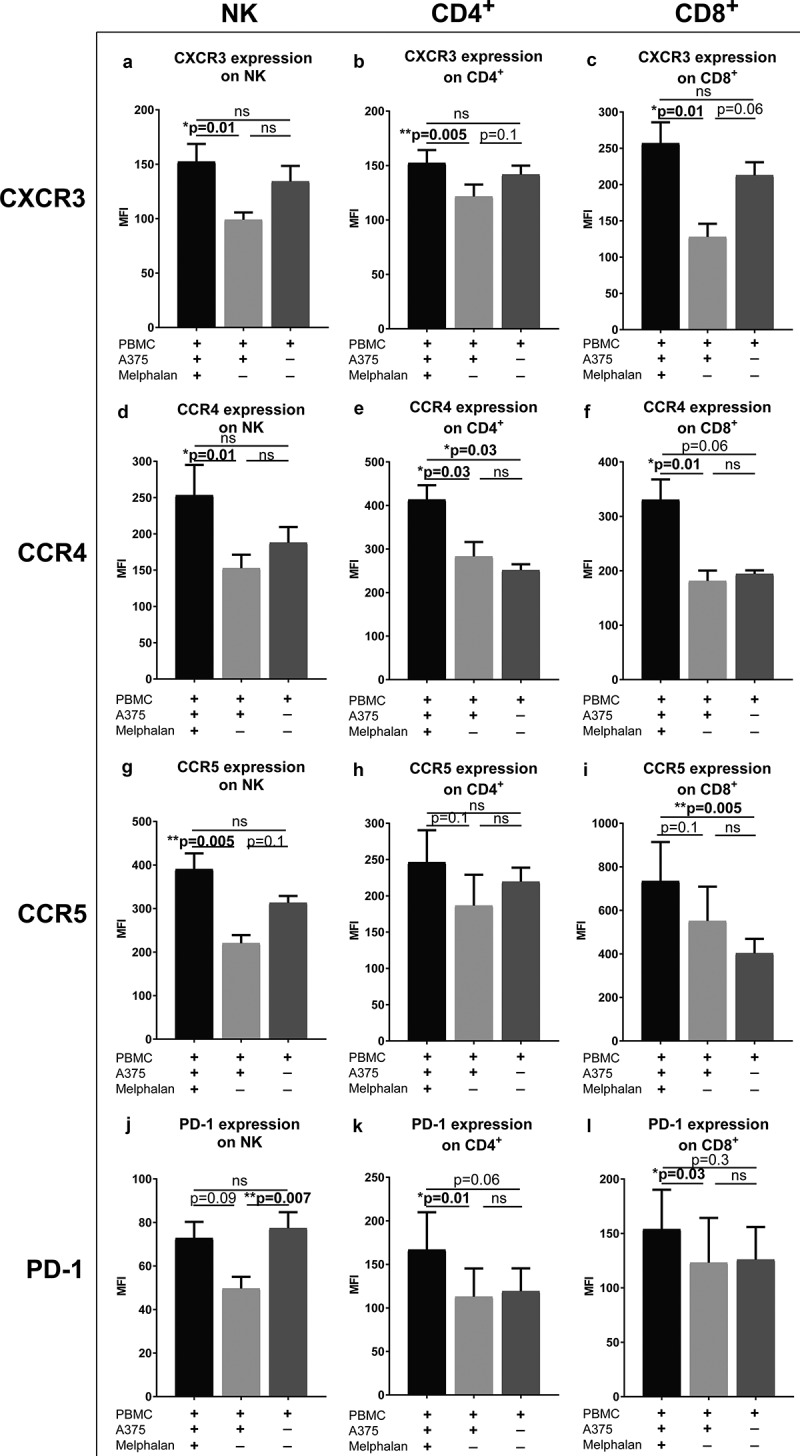
Melphalan-exposed melanoma cells induce expression of receptors for ISG products on PBMCs. PBMCs from healthy donors were cultured together with melphalan-exposed melanoma cells, non-exposed melanoma cells or were cultured alone. After 48 h, the PBMCs were transferred to new plates and were cultured in the absence of melanoma cells but presence of IL-2 for an additional 4 days. The expression of (a–c) CXCR3 (d-f) CCR4 (g–i) CCR5 and (j–l) PD-1 were measured on (a, d, g and j) NK cells (b, e, h and k) CD4^+^ T cells and (c, f, i and l) CD8^+^ T cells at the end of the culture (n = 6; paired Friedman test followed by Dunn’s multiple comparison test). MFI, Median Fluorescence Intensity. Data are presented as mean with SEM.

To determine effects of melphalan-exposed melanoma cells on lymphocyte expression of receptors for ISG products, non-adherent PBMCs were transferred to new plates after 48 h of co-culture followed by culture in medium with IL-2 for 4 days. Flow cytometry analysis revealed that CD4^+^ T cells, CD8^+^ T cells and NK cells expressed higher levels of CXCR3 and CCR4 after culture with melphalan-exposed melanoma cells than with non-exposed melanoma cells (Figure 6(a–f)). Similar results were obtained for CCR5 on NK cells and for PD-1 on CD4^+^ and CD8^+^ T cells (Figure 6(g–l)). As melanoma cells may express CXCR3, it was also investigated whether or not short-term exposure of melphalan induced CXCR3 expression on melanoma cells. In these experiments, melphalan-exposed A375 cells were found to significantly upregulate CXCR3 as compared with non-exposed control cells (Fig. S5).

### Activated t cells migrate toward supernatants from melphalan-exposed melanoma cells

**Figure 7.**
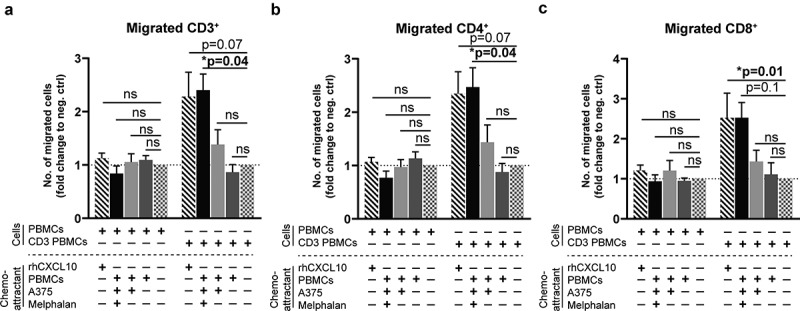
Activated T cells migrate toward supernatants from melphalan-exposed melanoma cells. PBMCs from healthy donors cultured for 4 days in IL-2 containing medium in the presence or absence of an anti-CD3 antibody (CD3 PBMCs; clone: OKT3) were used for chemotaxis experiments. The number of (a) CD3^+^, (b) CD4^+^ and (c) CD8^+^ T cells that migrated toward supernatants from PBMC-melanoma co-cultures was determined after 4 h of migration. Recombinant human CXCL10 (rhCXCL10) was used as a positive control, while medium was the negative control. (n = 6; Friedman test followed by Dunn’s multiple comparison test). The fractions of migrated T cells are presented as fold change compared to the negative control. Data are presented as mean with SEM.

To define the functional consequences of melphalan-induced formation of ISG-derived chemokines and their cognate receptors, we performed lymphocyte chemotaxis assays using supernatants obtained from the previously utilized PBMC–melanoma co-cultures. T cells that had been activated using an anti-CD3 antibody, but not non-activated T cells, showed significant migration toward supernatants derived from co-cultures of PBMCs and melphalan-exposed melanoma cells (Figure 7). The degree of induced migration was similar to that triggered by the positive control (recombinant human CXCL10). Supernatants obtained from co-cultures between PBMCs and non-melphalan exposed melanoma cells did not induce significant migration. In accordance, the presence of tumor-infiltrating lymphocytes (TILs) within ILP tumor biopsies, as determined by flow cytometry, correlated positively with the intratumoral levels of the chemokine CCL2, as measured in supernatants of single cell tumor biopsy suspensions (Fig. S6).

## Discussion

Patients with advanced cutaneous malignant melanoma may respond favorably to immunotherapy, such as neutralizing antibodies against CTLA-4 and PD-1/PD-L1.^17^ A high degree of immune cell infiltration, in particular by subsets of T cells, in the tumor microenvironment reportedly heralds favorable prognosis and responsiveness to checkpoint inhibitors, as reviewed in^18^ and.^19^ T cell infiltration in tumors may also predict a favorable response to chemotherapy and radiotherapy.^19,20^ Furthermore, chemotherapy and radiotherapy have been proposed to facilitate intratumoral infiltration of immune cells, thus enhancing the efficacy of checkpoint inhibitors.^20^

We have previously reported that circulating CD8^+^ T cells become activated following ILP and that the presence of activated CD8^+^ T cells in blood predicts complete disappearance of tumors in the perfused area.^13,14,21^ For the present study, we assessed the potential role of ISG-associated chemokines, chemokine receptors and related mediators for the benefit of ILP in patients with in-transit metastatic melanoma. We report that ILP patients who achieved CR showed higher serum levels of ISG products such as CXCL10, CCL2, PD-L2 and IFN-γ compared with non-CR patients. In agreement, analysis of TCGA data revealed a significant clinical benefit of high intratumoral mRNA expression of these ISGs in patients with advanced melanoma. While there were trends toward systemic induction of several ISG products after ILP, only levels of PD-L2 were significantly increased. In an *in vitro* model aiming to mimic ILP, we observed that melphalan exposure triggered a pronounced induction of CXCL10, CCL2 and IFN-γ in melanoma/PBMC co-cultures. In this setting, PBMCs constituted the main source of CXCL10, CCL2 and IFN-γ. IFN-α2 and IFN-β, were undetectable in these supernatants, thus suggesting that type I interferons were not major contributors to induction of ISG in these experiments.

We also observed that the expression of CXCR3, CCR4 and CCR5 was significantly higher on T cells and NK cells in peripheral blood of melanoma patients than in healthy controls. The lymphocyte expression of these receptors was further increased following ILP with similar results observed for PD-1 on CD4^+^ T cells, which is in agreement with a previous study.^13^ In analogy with these *in vivo* findings, results achieved using an *in vitro* model of ILP suggested that PBMCs cultured in the presence of melphalan-exposed melanoma cells expressed higher densities of CXCR3, CCR4, CCR5 and PD-1 than did PBMCs cultured with non-exposed melanoma cells. These results indicate that melphalan-exposed melanoma cells stimulate an ISG response comprising production of chemokines and other soluble mediators by immune cells along with induction of surface expression of chemokine receptors and PD-1 on lymphocytes.

The functional relevance of these findings was supported by results achieved in assays of T cell chemotaxis. Activated T cells (CD4^+^ and CD8^+^) thus showed significant migration toward supernatants from a PBMC co-culture with melphalan-exposed melanoma cells, but did not migrate significantly toward supernatants obtained from co-cultures of PBMCs and melanoma cells in the absence of melphalan. T cells that had not been activated with an anti-CD3 antibody, but merely expanded in the presence of IL-2 displayed no chemotactic capacity, which is in line with the view that the expression of chemokine receptors is restricted to activated or memory-type T cells.^22^ Overall, these findings support that the observed melphalan-induced induction of ISG-derived chemokines and their cognate receptor expression influences the chemotactic behavior of subsets of activated T cells.

We had access to melanoma tumors from patients obtained prior to ILP and observed a positive correlation between intratumoral CCL2 levels and CD8^+^ T cell content. Although these results were obtained in a small series of patients, the results are in line with previous studies indicating a link between ISG expression and presence of TILs in melanomas.^4,23^ When comparing receptor expression on TILs and PBMCs, the TILs showed higher expression of CCR5 and PD-1 but tended to express lower levels of CXCR3 and CCR4. While the limited number of biopsies available from ILP patients precluded a meaningful analysis of the potential clinical benefit of receptor-positive cells, the TCGA database showed significant overall survival benefit of high intratumoral expression of *CXCR3, CCR5* and *PDCD1* mRNA. In TCGA, the mRNA expression of *CCR4* did not significantly impact on survival, which may be explained by the predominant expression of CCR4 on T_h2_ cells and T_regs_, while CXCR3 and CCR5 are highly expressed by T_h1_ cells.^24^

CXCR3 has been ascribed a dual role in melanoma as its expression on lymphocytes may facilitate the recruitment of T cells and NK cells to the tumor microenvironment, but expression of CXCR3 on melanoma cells themselves may enhance metastatic spread.^8,9,25^ As we did not have access to tumor biopsies after ILP, *in vivo* effects of melphalan on CXCR3 expression on melanoma cells were not investigated. However, when comparing the expression of CXCR3 on melphalan-exposed and non-exposed melanoma cells *in vitro*, it was apparent that melphalan induced expression of CXCR3 on melanoma cells in addition to triggering its expression on lymphocytes. According to transcriptome analysis in the TCGA database, the net effect of high *CXCR3* expression in melanoma biopsies predicted favorable outcome in terms of overall survival.

In conclusion, the results of this study suggest that ILP triggers the transcription of ISG products, which may contribute to the regression of in-transit melanomas induced by this regimen. Further studies are warranted to define the impact of ISG induction on tumor regression following ILP, in addition to defining the potential impact of ILP on overall survival. We have recently initiated a clinical trial combining ILP and anti-PD1 antibodies (NCT03685890). The trial may shed further light on the potential role of ISGs and other aspects of immune activation for the clinical benefit of ILP.

## Materials and methods

### Patients and sampling of PBMCs and serum

Patients with in-transit melanoma metastases confined to a limb who received ILP at the Sahlgrenska University Hospital were included in this study. For patient characteristics, see Table S1. The ILP procedure is described in detail elsewhere.^10^ In brief, the blood circulation of the affected limb was isolated by cannulation of the major artery and vein under general anesthesia. The cannulas were then connected to an oxygenated extracorporeal circulation unit (heart-lung machine). Collateral vessels were compressed using an Esmarch bandage. Melphalan dosed according to limb volume (Alkeran®, 13 mg/l in upper limbs or 10 mg/l in lower limbs) was administered into the perfusion circuit during 20 min. The temperature was held at 40°C with a total perfusion time of 60 min. For patients with bulky tumors (>3 cm) or for patients undergoing a repeated procedure, TNF-α was also used, in addition to melphalan, during perfusion.

Peripheral blood was drawn approximately 1 day before and 1 month after ILP. PBMCs were purified from BD Vacutainer® CPT™ cell preparation tubes (BD Biosciences, #362782) according to the manufacturer’s protocol and subsequently cryopreserved. Peripheral blood was also collected in Vacuette® tubes (Greiner Bio-One, #455009) and serum was extracted after centrifugation for 10 min at 2000 g. Serum was aliquoted and frozen until analysis.

Tumor biopsies were obtained from patients before ILP. Single cell suspensions were prepared enzymatically and mechanically using the Tumor Dissociation Kit, human (Miltenyi Biotec, #130-095-929) and the gentleMACS™ Dissociator (Miltenyi Biotec) according to the manufacturer’s protocol. The cell suspensions were cryopreserved until analysis.

Clinical responses were evaluated according to the World Health Organization (WHO) criteria 3 months after ILP. Complete response (CR) was defined as disappearance of all lesions, partial response (PR) as reduction of more than 50% of the tumor burden, and progressive disease (PD) as an increase of more than 25% of existing lesions or the appearance of new lesions. Stable disease (SD) was defined by absence of the criteria for CR, PR or PD. For the analysis of ISG products and their receptors vs. outcome, clinical responses were defined as either CR or non-CR (i.e. PR, PD and SD). All patients gave written consent and the study was approved by the Regional Ethical Review Board in Gothenburg, Sweden (No. 424–14).

### Sampling of PBMCs and serum from healthy controls

PBMCs were purified from buffy coats from anonymous healthy donors obtained from the Component Laboratory at Sahlgrenska University Hospital. The PBMCs were isolated with dextran sedimentation followed by density gradient separation with Lymphoprep™ (Alere Technologies AS, #1114547). Serum was extracted from peripheral blood from healthy donors as described above.

### Culture of tumor single cell suspensions

Tumor single cell suspensions were thawed and seeded in U-bottomed 96-well plates at seeding densities of 20 × 10^4^ cells/ml or 100 × 10^4^ cells/ml. The cells were cultured in IMDM (ThermoFisher Scientific, #21980065) supplemented with 10% heat-inactivated fetal bovine serum (Sigma-Aldrich) and 100 U/ml penicillin-streptomycin (ThermoFisher Scientific, #15140122) for 48 h at 37°C and 5% CO_2_. At the end of the culture, cell culture supernatants were collected and frozen, while RNA was isolated from cells using the RNeasy Plus Mini Kit (Qiagen, #74134) according to manufacturer’s protocol. Potential contamination by melanin was removed with RNeasy PowerClean Pro Cleanup Kit (Qiagen, #13997-50).

### In vitro model of hyperthermic isolated limb perfusion

An *in vitro* model of ILP was established using the human melanoma cell line A375 and PBMCs from healthy donors. A375 cells were purchased from CLS Cell Lines Service GmbH (Eppelheim, Germany) and authenticated using Multiplex Cell Authentication by Multiplexion (Heidelberg, Germany) as described.^26^ The SNP profile matched the known profile. The cell line was tested for mycoplasma contamination using PCR at the Bacteriology Laboratory at Sahlgrenska University Hospital (Gothenburg, Sweden). A375 cells were cultured in DMEM (Sigma-Aldrich, #D6429) supplemented with 10% heat-inactivated fetal bovine serum, 100 U/ml penicillin-streptomycin, 10 μg/ml Fungin™ (InvivoGen, #ant-fn-1), 1 mM sodium pyruvate (Sigma-Aldrich, #S8636) and 2 mM L-glutamine (Life Technologies, #25030123) at 37°C and 5% CO_2_. The cells were exposed to a sub-lethal concentration (50 µM, causing 20–30% cell death) of melphalan hydrochloride (Alkeran®) for 1 h aiming to mimic the clinical ILP protocol. After exposure, the melanoma cells were thoroughly washed and cultured in complete medium overnight after which PBMCs from healthy donors were added to the melanoma cells in co-culture. PBMCs were cultured with melphalan-exposed (or non-exposed) A375 cells in flat-bottomed 48 well-plates for 48 h. Thereafter, the supernatant was collected and the non-adherent PBMCs were transferred to new cell culture plates for cultivation for an additional 4 days in IMDM with 10% heat-inactivated fetal bovine serum, 100 U/ml penicillin-streptomycin, 10 μg/ml Fungin™, 2 mM L-glutamine and 500 U/ml recombinant human IL-2 (PeproTech, #200-02).

### Chemotaxis assay

PBMCs from healthy donors were cultured for 4 days in IMDM with 10% heat-inactivated fetal bovine serum, 100 U/ml penicillin-streptomycin, 10 μg/ml Fungin™, 2 mM L-glutamine, 1 mM sodium pyruvate and 500 U/ml recombinant human IL-2. Half of the cultures were also supplemented with an anti-CD3 monoclonal antibody (clone: OKT3, 50 ng/ml, eBioscience™, #16-0037-85) in order to activate the T cells. After 4 days, the PBMCs were resuspended in IMDM with 0.1% bovine serum albumin (MP Biomedical, #160069) at a concentration of 1 × 10^6^ cells/ml, and 100 μl of the cell suspension was added to Transwell® inserts with pore size 5.0 μm placed in flat-bottomed 24-well plates (Corning® Transwell® polycarbonate membrane cell culture inserts, Sigma-Aldrich, #CLS3421-48EA). IMDM with 10% heat-inactivated fetal bovine serum, with or without recombinant human CXCL10 (100 ng/ml, R&D Systems, #266-IP) as a positive control or supernatants obtained from PBMC-melanoma co-cultures collected 48 h after start of the co-culture (two parts supernatant and one part complete IMDM medium), was added to the wells underneath the Transwell® inserts. After 4 h of incubation at 37°C, the migrated cells were collected, stained with T cell and NK cell markers and counted using counting beads (CountBright™ Absolute Counting Beads, Thermo Fisher Scientific, #C36950) by flow cytometry. The fraction of cells that migrated was calculated as fold change to the negative control, e.g. IMDM with only 10% serum and no additional chemoattractant.

### Analysis of chemokines and other soluble factors in serum and culture supernatants

Chemokines and other soluble factors from serum were assessed in multiplex immunoassays utilizing Luminex technology. Serum from ILP patients and from healthy donors were analyzed with a 14-plex immune checkpoint panel (Human Immuno-Oncology Checkpoint Marker Panel, Invitrogen, #EPX14A-15803-901) and a 27-plex cytokine panel (Bio-Plex™ Pro Human Cytokine Standard 27-Plex Group 1, Bio-Rad, #M500KCAF0Y) according to the manufacturers’ protocols. All measurements were performed using a Bio-Plex™ 200 System (Bio-Rad).

Soluble CXCL10, CCL2, IFN-γ, IFN-α2 and IFN-β in cell culture supernatants were measured using a CXCL10 ELISA kit (Human CXCL10/IP-10 DuoSet ELISA, R&D Systems, #DY266-05), a CCL2 ELISA kit (Human CCL2/MCP-1 DuoSet ELISA, R&D Systems, #DY279-05) and ELISA kits for IFN-γ, IFN-α2 and IFN-β (Human IFN-gamma/IFN-alpha 2/IFN-beta DuoSet ELISA, R&D Systems, #DY285B-05/#DY9345-05/#DY814-05) together with Substrate Reagent Pack (R&D Systems, #DY999) in 96-well plates (Corning™ 96-Well Half-Area Plates, Fisher Scientific, #10052511) according to manufacturer’s protocol. For samples with protein levels lower than the detection limit, 50% of the lowest detected concentration was utilized in statistical calculations.

### Flow cytometry

Staining with conjugated antibodies was performed in phosphate buffered saline (PBS) with 0.5% BSA and 0.1% EDTA. Cell viability was determined using LIVE/DEAD™ Fixable Yellow Dead Cell Stain Kit (Life Technologies, #L34959). Table S2 provides a list of conjugated antibodies. Flow cytometry was conducted on a BD LSRFortessa™ instrument (BD Biosciences) with subsequent data analysis using BD FACSDiva Software version 8.0.1 (BD Biosciences).

### Reverse transcription quantitative PCR (RT-qPCR)

RNA from tumor biopsy cultures were reverse transcribed into cDNA using the TATAA GrandScript cDNA Synthesis Kit (TATAA Biocenter, #A103b) at 22°C for 5 min, 42°C for 30 min, and 85°C for 5 min. Gene expression was determined with qPCR utilizing TATAA SYBR® GrandMaster® Mix (TATAA Biocenter, # TA01-5000) in a CFX384 Touch Real-Time PCR Detection System (Bio-Rad), with a thermal cycling profile of 95°C for 2 min followed by 50 cycles of amplification (95°C for 5 s, 60°C for 20 s, 70°C for 20 s). Cycle of quantification (Cq) values were determined with regression. Pre-processing was performed in GenEx ver. 6 (MultiD Analyses AB). Briefly, missing data were replaced with Cq values corresponding to one-fourth of the minimum number of molecules detected in the assay and all data were normalized to the reference gene *GAPDH*, chosen among eight potential reference genes with the NormFinder algorithm. Cq values were converted to relative quantities and technical replicates were averaged. qPCR primers were ordered from Sigma-Aldrich, and a full list of the primers is found in Table S3.

### Statistical analyses

Statistical analyses were performed in GraphPad Prism 8.0.2 (GraphPad Software) and non-parametric statistical tests were utilized for all samples except for the experiment involving only cell lines (Fig. S2). To test for multiple comparisons Kruskal-Wallis test was used for non-paired samples and Friedman test was used for paired samples, both followed by Dunn’s multiple comparison test. Single comparisons were made with paired Wilcoxon and paired t-test. Fisher’s exact test was used to compare distribution within two groups. Survival analyses were performed by Kaplan-Meier statistics and log-rank tests. Correlation analyses were performed by Spearman correlation.

## References

[1] Lee AJ, Ashkar AA. The dual nature of type I and type II interferons. Front Immunol. 2018;9:2061. doi:10.3389/fimmu.2018.02061. 30254639 PMC6141705

[2] Darnell JE Jr., Kerr IM, Stark GR. Jak-STAT pathways and transcriptional activation in response to IFNs and other extracellular signaling proteins. Science. 1994;264(5164):1415–12. doi:10.1126/science.8197455. 8197455

[3] de Veer MJ, Holko M, Frevel M, Walker E, Der S, Paranjape JM, Silverman RH, Williams BR. Functional classification of interferon-stimulated genes identified using microarrays. J Leukoc Biol. 2001;69(6):912–920. 11404376

[4] Harlin H, Meng Y, Peterson AC, Zha Y, Tretiakova M, Slingluff C, McKee M, Gajewski TF. Chemokine expression in melanoma metastases associated with CD8+ T-cell recruitment. Cancer Res. 2009;69(7):3077–3085. doi:10.1158/0008-5472.CAN-08-2281. 19293190 PMC3886718

[5] Luster AD, Unkeless JC, Ravetch JV. Gamma-interferon transcriptionally regulates an early-response gene containing homology to platelet proteins. Nature. 1985;315(6021):672–676. doi:10.1038/315672a0. 3925348

[6] Loetscher M, Gerber B, Loetscher P, Jones SA, Piali L, Clark-Lewis I, Baggiolini M, Moser B. Chemokine receptor specific for IP10 and mig: structure, function, and expression in activated T-lymphocytes. J Exp Med. 1996;184(3):963–969. doi:10.1084/jem.184.3.963. 9064356 PMC2192763

[7] Qin S, Rottman JB, Myers P, Kassam N, Weinblatt M, Loetscher M, Koch AE, Moser B, Mackay CR. The chemokine receptors CXCR3 and CCR5 mark subsets of T cells associated with certain inflammatory reactions. J Clin Invest. 1998;101(4):746–754. doi:10.1172/JCI1422. 9466968 PMC508621

[8] Kawada K, Sonoshita M, Sakashita H, Takabayashi A, Yamaoka Y, Manabe T, Inaba K, Minato N, Oshima M, Taketo MM. Pivotal role of CXCR3 in melanoma cell metastasis to lymph nodes. Cancer Res. 2004;64(11):4010–4017. doi:10.1158/0008-5472.CAN-03-1757. 15173015

[9] Jenkins MH, Brinckerhoff CE, Mullins DW, Chen S. CXCR3 signaling in BRAFWT melanoma increases IL-8 expression and tumorigenicity. PLoS One. 2015;10(3):e0121140. doi:10.1371/journal.pone.0121140. 25798946 PMC4370421

[10] Olofsson R, Mattsson J, Lindner P. Long-term follow-up of 163 consecutive patients treated with isolated limb perfusion for in-transit metastases of malignant melanoma. Int J Hyperthermia. 2013;29(6):551–557. doi:10.3109/02656736.2013.802374. 23865737

[11] Moreno-Ramirez D, de la Cruz-merino L, Ferrandiz L, Villegas-Portero R, Nieto-Garcia A. Isolated limb perfusion for malignant melanoma: systematic review on effectiveness and safety. Oncologist. 2010;15(4):416–427. doi:10.1634/theoncologist.2009-0325. 20348274 PMC3227960

[12] Testori A, Verhoef C, Kroon HM, Pennacchioli E, Faries MB, Eggermont AMM, Thompson JF. Treatment of melanoma metastases in a limb by isolated limb perfusion and isolated limb infusion. J Surg Oncol. 2011;104(4):397–404. doi:10.1002/jso.22028. 21858835

[13] Johansson J, Kiffin R, Andersson A, Lindnér P, Naredi PL, Olofsson Bagge R, Martner A. Isolated limb perfusion with melphalan triggers immune activation in melanoma patients. Front Oncol. 2018;8:570. doi:10.3389/fonc.2018.00570. 30560089 PMC6286961

[14] Olofsson R, Lindberg E, Karlsson-Parra A, Lindnér P, Mattsson J, Andersson B. Melan-A specific CD8+ T lymphocytes after hyperthermic isolated limb perfusion: a pilot study in patients with in-transit metastases of malignant melanoma. Int J Hyperthermia. 2013;29(3):234–238. doi:10.3109/02656736.2013.782428. 23590363

[15] Wieberdink J, Benckhuysen C, Braat RP, Van Slooten EA, Olthuis GAA. Dosimetry in isolation perfusion of the limbs by assessment of perfused tissue volume and grading of toxic tissue reactions. Eur J Cancer Clin Oncol. 1982;18(10):905–910. doi:10.1016/0277-5379(82)90235-8. 6891640

[16] Yu N, Li X, Song W, Li D, Yu D, Zeng X, Li M, Leng X, Li X CD4(+)CD25 (+)CD127 (low/-) T cells: a more specific Treg population in human peripheral blood. Inflammation. 2012;35(6):1773–1780. doi:10.1007/s10753-012-9496-8. 22752562

[17] Hodi FS, Chiarion-Sileni V, Gonzalez R, Grob -J-J, Rutkowski P, Cowey CL, Lao CD, Schadendorf D, Wagstaff J, Dummer R. Nivolumab plus ipilimumab or nivolumab alone versus ipilimumab alone in advanced melanoma (CheckMate 067): 4-year outcomes of a multicentre, randomised, phase 3 trial. Lancet Oncol. 2018;19(11):1480–1492. doi:10.1016/S1470-2045(18)30700-9. 30361170

[18] Ladanyi A. Prognostic and predictive significance of immune cells infiltrating cutaneous melanoma. Pigment Cell Melanoma Res. 2015;28(5):490–500. doi:10.1111/pcmr.12371. 25818762

[19] Barnes TA, Amir E. HYPE or HOPE: the prognostic value of infiltrating immune cells in cancer. Br J Cancer. 2017;117(4):451–460. doi:10.1038/bjc.2017.220. 28704840 PMC5558691

[20] Zitvogel L, Kepp O, Kroemer G. Immune parameters affecting the efficacy of chemotherapeutic regimens. Nat Rev Clin Oncol. 2011;8(3):151–160. doi:10.1038/nrclinonc.2010.223. 21364688

[21] Martner A, Johansson J, Ben-Shabat I, Olofsson-Bagge R. Melphalan, antimelanoma immunity, and inflammation–letter. Cancer Res. 2015;75(24):5398–5399. doi:10.1158/0008-5472.CAN-15-1184. 26627012

[22] Mackay CR. Chemokine receptors and T cell chemotaxis. J Exp Med. 1996;184(3):799–802. doi:10.1084/jem.184.3.799. 9064339 PMC2192795

[23] Linsley PS, Speake C, Whalen E, Chaussabel D. Copy number loss of the interferon gene cluster in melanomas is linked to reduced T cell infiltrate and poor patient prognosis. PLoS One. 2014;9(10):e109760. doi:10.1371/journal.pone.0109760. 25314013 PMC4196925

[24] Bonecchi R, Bianchi G, Bordignon PP, D'Ambrosio D, Lang R, Borsatti A, Sozzani S, Allavena P, Gray PA, Mantovani A, et al. Differential expression of chemokine receptors and chemotactic responsiveness of type 1 T helper cells (Th1s) and Th2s. J Exp Med. 1998;187(1):129–134. doi:10.1084/jem.187.1.129. 9419219 PMC2199181

[25] Monteagudo C, Martin JM, Jorda E, Llombart-Bosch A. CXCR3 chemokine receptor immunoreactivity in primary cutaneous malignant melanoma: correlation with clinicopathological prognostic factors. J Clin Pathol. 2007;60(6):596–599. doi:10.1136/jcp.2005.032144. 16522748 PMC1955073

[26] Castro F, Dirks WG, Fähnrich S, Hotz-Wagenblatt A, Pawlita M, Schmitt M. High-throughput SNP-based authentication of human cell lines. Int J Cancer. 2013;132(2):308–314. 22700458 10.1002/ijc.27675 PMC3492511

