# Supplementary material for: Isolated limb perfusion with melphalan activates interferon-stimulated genes to induce tumor regression in patients with melanoma in-transit metastasis
Source: Oncoimmunology. 2019 Nov 3;9(1):1684126. doi: 10.1080/2162402X.2019.1684126 (PMC6959433; doi:10.1080/2162402X.2019.1684126)
Supplement: Supplemental Material [file koni-09-01-1684126-s001.pdf]

## SUPPLEMENTARY MATERIALS

| | |
|---|---|
| Sex | |
| Female | 35 (51%) |
| Male | 33 (49%) |
| Age (median + range) | 73 years (40-87) |
| Clinical response | |
| CR | 38 (56%) |
| PR | 14 (21%) |
| SD | 4 (6%) |
| PD | 8 (12%) |
| N/A | 4 (6%) |
| N stage | |
| N0 | 1 (1%) |
| N1 | 1 (1%) |
| N2 | 1 (1%) |
| N2c | 37 (54%) |
| N3 | 28 (41%) |
| M stage | |
| M0 | 62 (91%) |
| N/A | 6 (9%) |
| Perfusion vessel | |
| Brachial | 9 (13%) |
| Femoral | 59 (87%) |
| Perfusion chemo | |
| Melphalan | 59 (87%) |
| Melphalan + TNF-α | 9 (13%) |

**Table S1**. Patient and tumor characteristics.

| Antigen | Fluorophore | Clone | Vendor | Article number |
|---|---|---|---|---|
| CCR4 | BV421 | 1G1 | BD Biosciences | 562579 |
| CCR5 | FITC | 2D7 | BD Biosciences | 561747 |
| CD127 | Alexa Fluor® 647 | HIL-7R-M21 | BD Biosciences | 558598 |
| CD25 | PE | M-A251 | BD Biosciences | 555432 |
| CD3 | BV711 | UCHT1 | BD Biosciences | 563725 |
| CD4 | APC-H7 | RPA-T4 | BD Biosciences | 560158 |
| CD8 | PerCP-Cy5.5 | RPA-T8 | BD Biosciences | 560662 |
| CD56 | BUV737 | NCAM16.2 | BD Biosciences | 564447 |
| CXCR3 | BUV395 | 1C6 | BD Biosciences | 565223 |
| PD-1 | BV421 | EH12.1 | BD Biosciences | 562516 |

**Table S2**. List of all conjugated antibodies for flow cytometry utilized in the study.

| Gene | Forward primer | Reverse primer |
|---|---|---|
| *CCL2* | TCAAACTGAAGCTCGCACTCT | GGCATTGATTGCATCTGGC |
| *CXCL10* | AGCAGTTAGCAAGGAAAGGTCTA | GGGAAGTGATGGGAGAGGCA |
| *GAPDH* | CCCACTCCTCCACCTTTGAC | GCCAAATTCGTTGTCATACCAGG |
| *PDCD1LG2* | CGTGAGGGAACTTACTTTGGC | GCAGAAGGGGATGAAAATGTGA |

**Table S3.** Primers for RT-qPCR.

**SUPPLEMENTARY FIGURES**

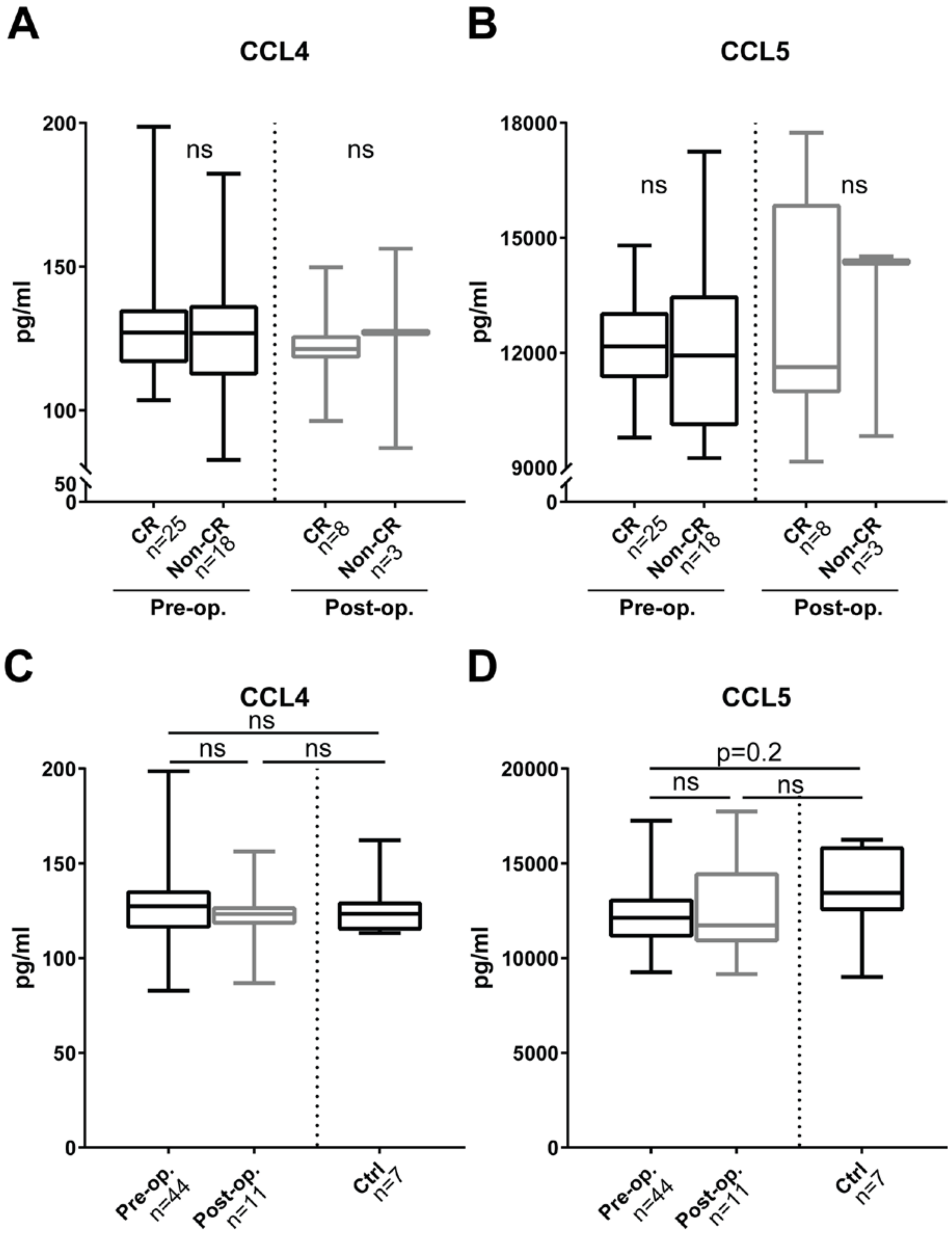


**Figure S1. Serum levels of CCL4 and CCL5 in melanoma patients and healthy controls.**
Serum levels of **A, C)** CCL4 and **B, D)** CCL5 in **A-B)** melanoma patients who achieved and did not achieve CR (non-paired Mann-Whitney test), and **C-D)** between melanoma patients and healthy controls (paired Wilcoxon test between pre-op. and post-op., non-paired Kruskal-Wallis test followed by Dunn's multiple comparison test for ctrl vs. pre-op. and vs. post-op). Pre-op. indicates blood sample obtained before ILP, post-op. indicates samples obtained one month after ILP. Data are presented in box-and-whiskers plots with min. and max.

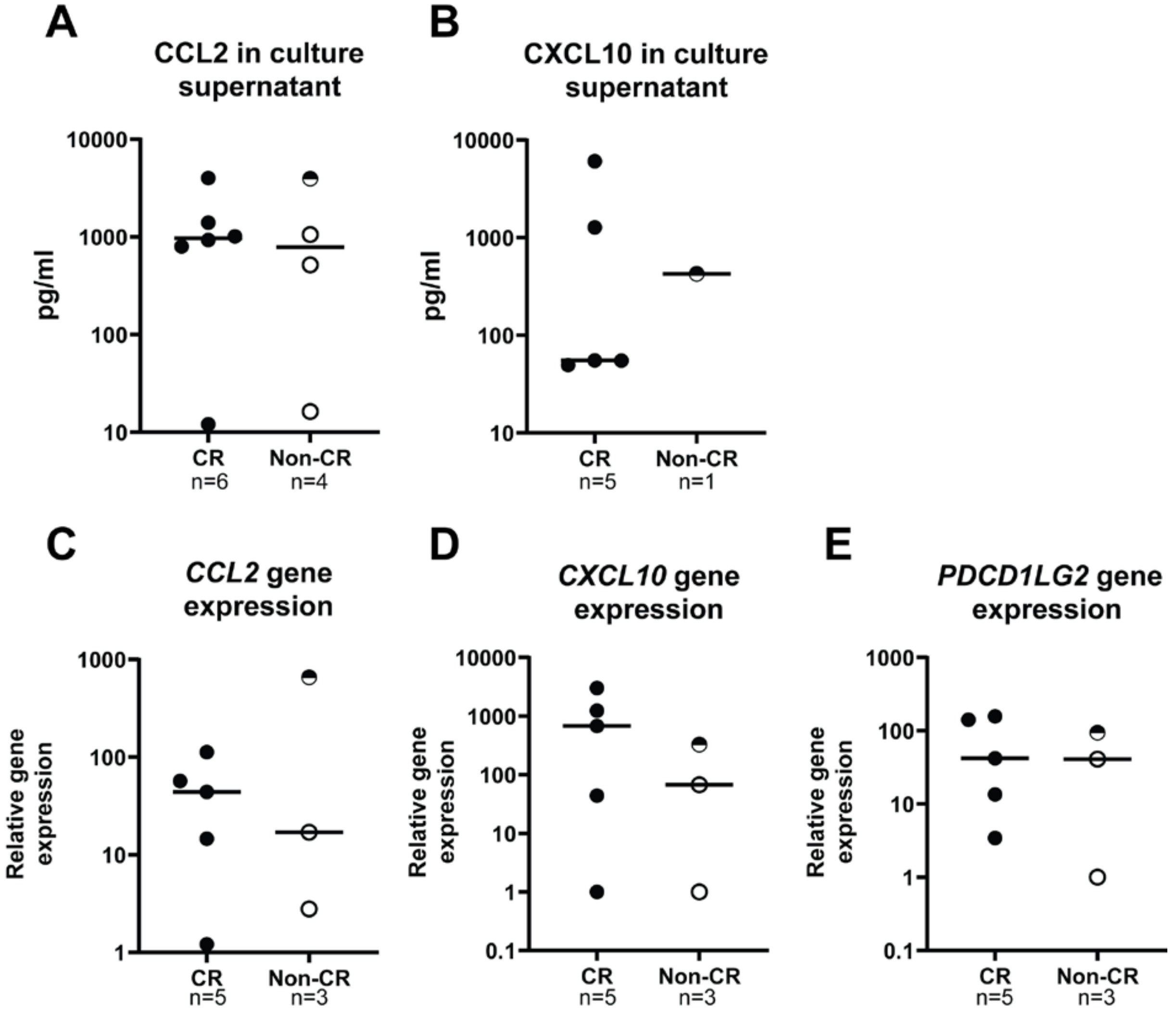


**Figure S2. Correlation between treatment response and intratumoral levels of ISGs and ISG products.**

Levels of **A)** CCL2 and **B)** CXCL10 in tumor biopsy culture supernatants from ILP patients stratified into CR and Non-CR. Biopsies were obtained during the operational procedure. mRNA was extracted from the same biopsy cultures, and subsequent RT-qPCR analyzes were made with primers for **C)** *CCL2*, **D)** *CXCL10* and **E)** *PDCD1LG2*. RT-qPCR data were normalized to the reference gene *GAPDH*, converted to relative units and stratified into CR or Non-CR. Among the Non-CR patients one patient was a partial responder (PR) and is represented by a half-filled circle.

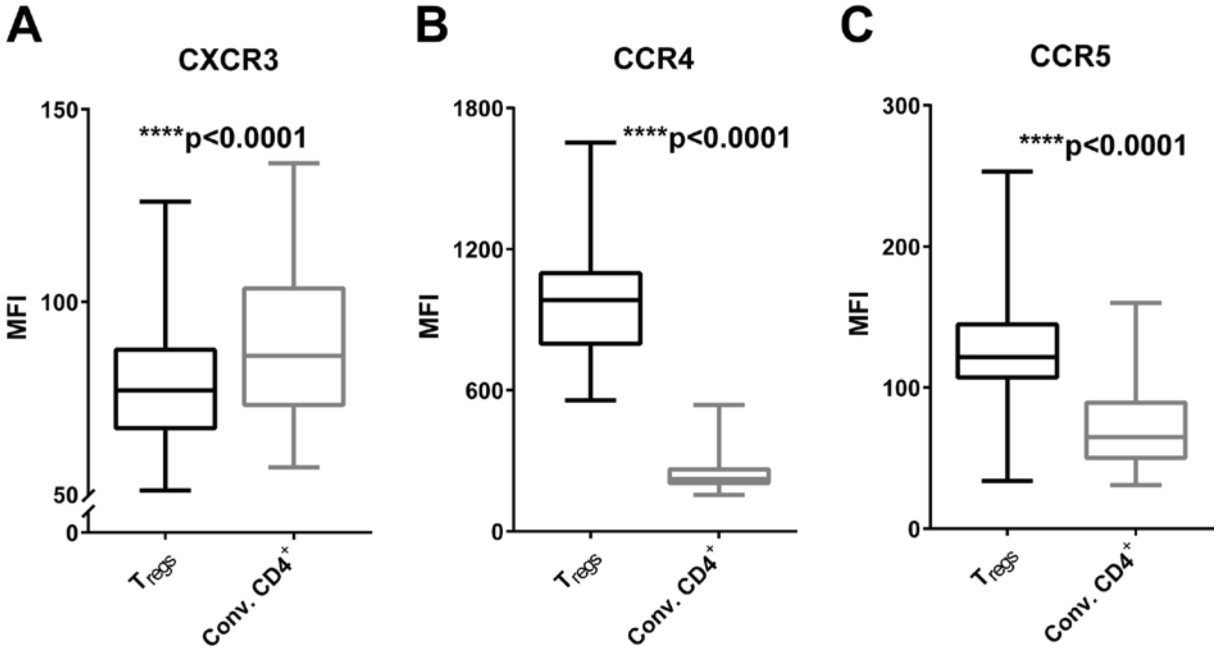


**Figure S3. $T_{regs}$ and conventional $CD4^+$ T cells express different levels of chemokine receptors.** The expression of **A)** CXCR3, **B)** CCR4 and **C)** CCR5 on $T_{regs}$ and conventional $CD4^+$ T cells in peripheral blood from melanoma patients before ILP (n=54; paired Wilcoxon test). MFI = Median Fluorescence Intensity. Data are presented in box-and-whiskers plots with min. and max.

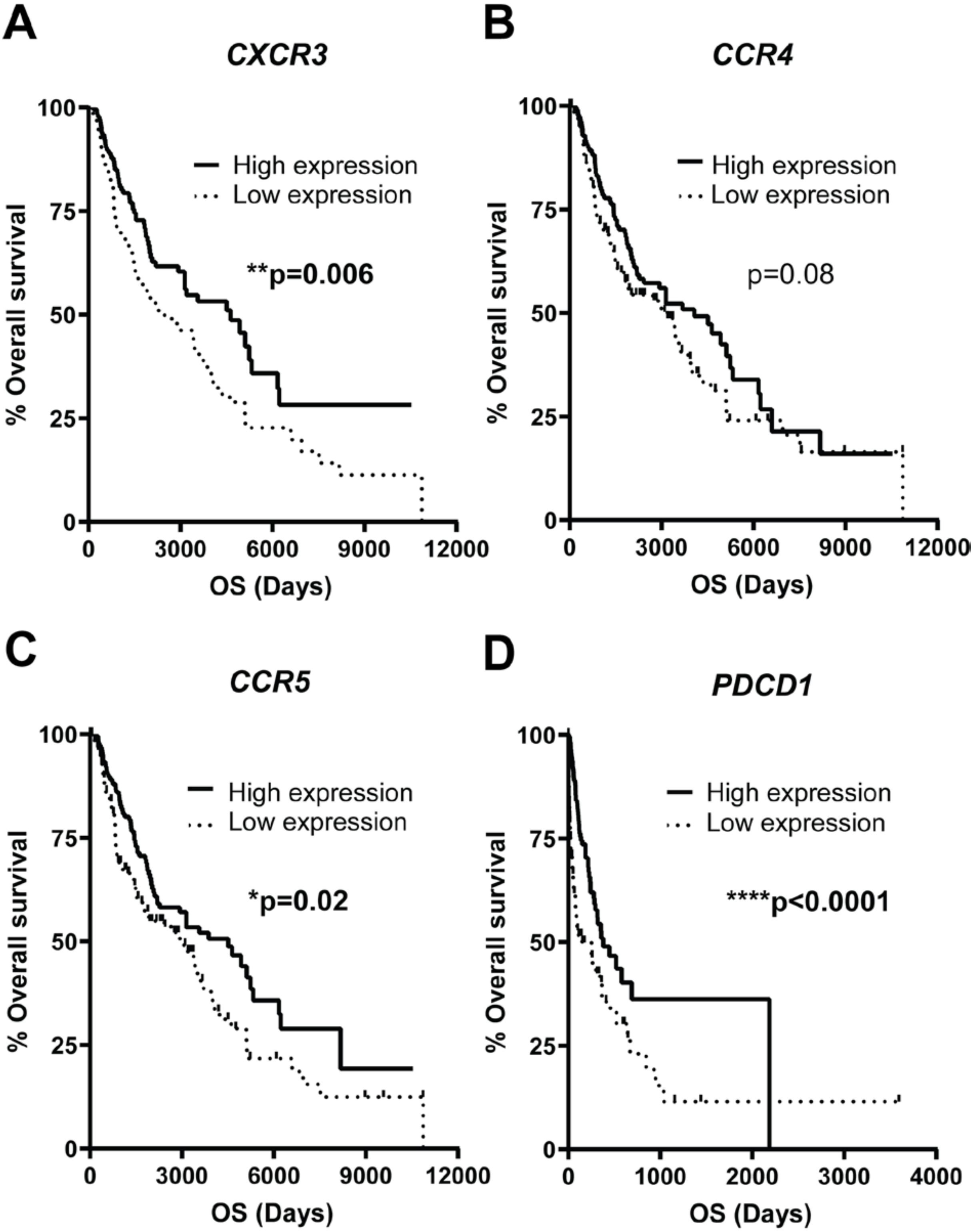


**Figure S4. High mRNA expression of receptors to ISG products correlates with prolonged overall survival in advanced melanoma.**
Melanoma patients in the TCGA database were dichotomized into two groups based on above or below median gene expression of the receptors **A)** *CXCR3*, **B)** *CCR4*, **C***)* *CCR5* and **D)** *PDCD1* (n=470; Log-rank test).

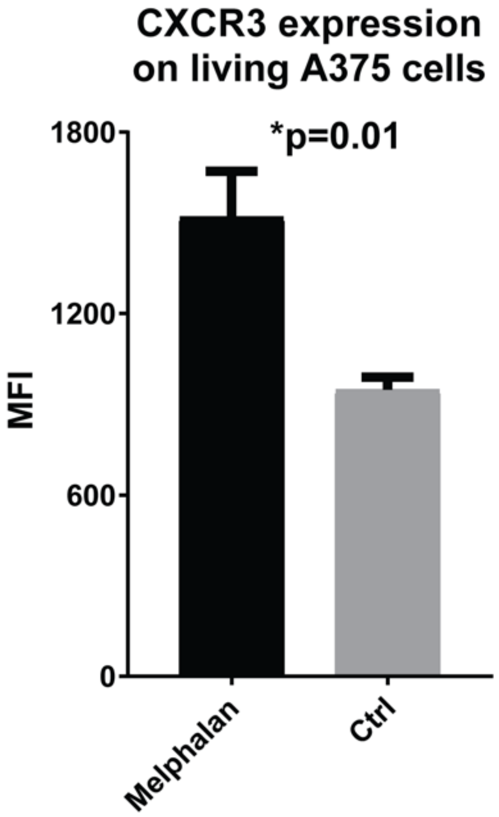


**Figure S5. Melanoma cells exposed to melphalan upregulates CXCR3.**
The expression of CXCR3 on melphalan-exposed and non-exposed melanoma cells (n=5; paired t-test). MFI = Median Fluorescence Intensity. Data are presented as mean with SEM.

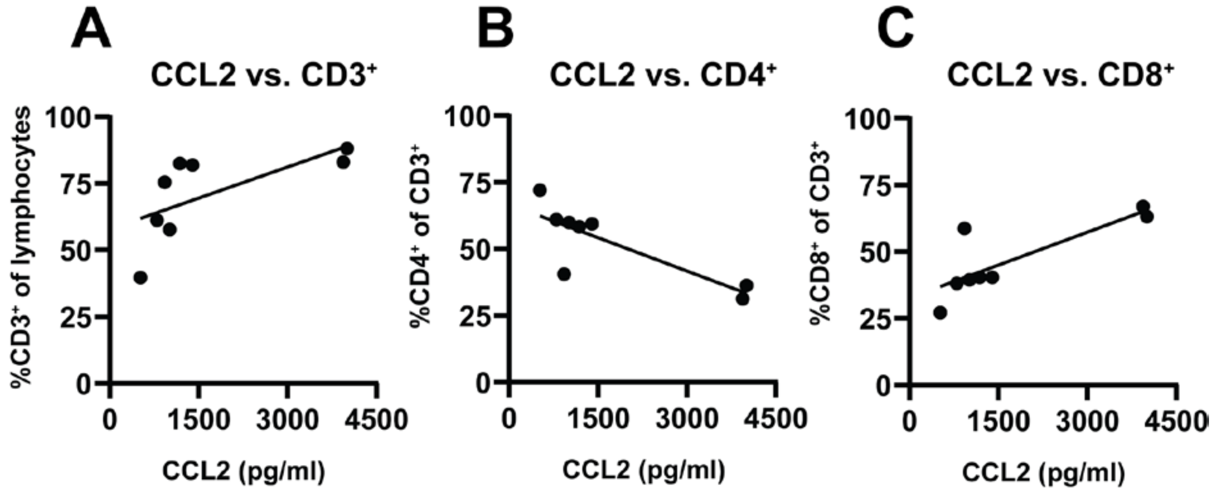


**D**

**Intratumoral levels of CCL2 and percent TILs**

| | | %CD3⁺ of lymphocytes | %CD4⁺ of CD3⁺ | %CD8⁺ of CD3⁺ |
|---|---|---|---|---|
| **CCL2 vs.** | Spearman r | 0.91 | -0.81 | 0.83 |
| | P value | **0.0046 | *0.022 | *0.016 |

**Figure S6. High levels of CCL2 in tumors correlate with infiltration of T cells.**
Tumor biopsies were obtained during ILP and processed into single cell suspensions. TILs were obtained and analyzed with FACS, and supernatants were collected after 48 hours of culture of the cell suspensions and analyzed for the presence of CCL2. The level of CCL2 is depicted against the percentage of **D)** $CD3^+$ T cells among lymphocytes, **E)** $CD4^+$ and **F)** $CD8^+$ T cells among $CD3^+$ T cells, with the lines adapted from linear regression. **G)** The level of CCL2 was correlated against the T cell populations utilizing Spearman correlation. (n=8)
